# Synchronization-Induced Rhythmicity of Circadian Oscillators in the Suprachiasmatic Nucleus

**DOI:** 10.1371/journal.pcbi.0030068

**Published:** 2007-04-13

**Authors:** Samuel Bernard, Didier Gonze, Branka Čajavec, Hanspeter Herzel, Achim Kramer

**Affiliations:** 1 Institute of Applied and Computational Mathematics, Foundation for Research and Technology–Hellas, Heraklion, Crete, Greece; 2 Unité de Chronobiologie Théorique, Faculté des Sciences, CP 231, Université Libre de Bruxelles, Bruxelles, Belgium; 3 Institute for Theoretical Biology, Humboldt University, Berlin, Germany; 4 Laboratory of Chronobiology, Institute of Medical Immunology, Charité Universitätsmedizin Berlin, Berlin, Germany; University College London, United Kingdom

## Abstract

The suprachiasmatic nuclei (SCN) host a robust, self-sustained circadian pacemaker that coordinates physiological rhythms with the daily changes in the environment. Neuronal clocks within the SCN form a heterogeneous network that must synchronize to maintain timekeeping activity. Coherent circadian output of the SCN tissue is established by intercellular signaling factors, such as vasointestinal polypeptide. It was recently shown that besides coordinating cells, the synchronization factors play a crucial role in the sustenance of intrinsic cellular rhythmicity. Disruption of intercellular signaling abolishes sustained rhythmicity in a majority of neurons and desynchronizes the remaining rhythmic neurons. Based on these observations, the authors propose a model for the synchronization of circadian oscillators that combines intracellular and intercellular dynamics at the single-cell level. The model is a heterogeneous network of circadian neuronal oscillators where individual oscillators are damped rather than self-sustained. The authors simulated different experimental conditions and found that: (1) in normal, constant conditions, coupled circadian oscillators quickly synchronize and produce a coherent output; (2) in large populations, such oscillators either synchronize or gradually lose rhythmicity, but do not run out of phase, demonstrating that rhythmicity and synchrony are codependent; (3) the number of oscillators and connectivity are important for these synchronization properties; (4) slow oscillators have a higher impact on the period in mixed populations; and (5) coupled circadian oscillators can be efficiently entrained by light–dark cycles. Based on these results, it is predicted that: (1) a majority of SCN neurons needs periodic synchronization signal to be rhythmic; (2) a small number of neurons or a low connectivity results in desynchrony; and (3) amplitudes and phases of neurons are negatively correlated. The authors conclude that to understand the orchestration of timekeeping in the SCN, intracellular circadian clocks cannot be isolated from their intercellular communication components.

## Author Summary

Circadian rhythms, characterized by a period close to 24 h, are observed in nearly all living organisms, from cyanobacteria to plants, insects, and mammals. In mammals, the central circadian clock is located in the suprachiasmatic nucleus (SCN) of the hypothalamus, where it receives light signals from the retina. In turn, the SCN controls circadian rhythms in peripheral tissues and behavioral activity. The SCN is composed of about 20,000 neurons characterized by a small size and a high density. Within each individual neuron, clock genes and proteins compose interlocked regulatory feedback loops that generate circadian oscillations on the molecular level. SCN neurons dispersed in cell cultures display cell-autonomous oscillations, with periods ranging from 20 h to 28 h. The ventrolateral part of the SCN receives light input from the retina, serving as a relay for the dorsomedial part. Coupling and synchronization among SCN neurons are ensured by neurotransmitters. A desire to understand how such a network of heterogeneous circadian oscillators achieves a synchronous and coherent output rhythm has motivated extensive experimental and theoretical work. In this paper, we present a molecular model combining intracellular and extracellular dynamics for the SCN circadian system, and propose a novel synchronization mechanism. Our results predict a dual role for the coupling factors within the SCN, both in maintaining the rhythmicity and in promoting the synchronization between the circadian oscillators.

## Introduction

In most mammalian cells, a set of “clock” genes and proteins forms a regulatory network that produces oscillations with a circadian period (≈24 h) [1]. Molecular and physiological rhythms are coordinated with the daily changes in the environment by a dominant circadian pacemaker, the suprachiasmatic nuclei (SCN) of the hypothalamus. The SCN neurons endogenously generate circadian rhythm and adapt that rhythm according to light–dark (LD) cycles of the environment (entrainment). The approximately 20,000 neurons in the SCN [2,3] vary (1) in their ability to sense the environmental timing cues, (2) in the neurotransmitters they express or respond to, and (3) in their connectivity properties. A desire to understand how such a heterogeneous network produces a coherent and synchronous circadian output has motivated extensive experimental and theoretical work.

Organotypic SCN slices or SCN neurons in high-density dispersal cultures express a coordinated rhythmic activity for as long as they are viable (a few weeks up to several months) [2]. SCN neurons in low-density dispersal cultures, however, do not show a coordinated activity but express a large variation in their free-running periods [4,5]. This has led to the conclusion that SCN neurons are self-sustained circadian oscillators that need a synchronization signal to produce a coherent output. Even before this experimental evidence, it had been hypothesized that the coupling of “sloppy” clocks improves the reliability of the output [6]. So far, all published mathematical models of the synchronization of the SCN rest on the coupling of self-sustained circadian oscillators.

Among candidate synchronization factors are the neuropeptides vasoactive intestinal polypeptide (VIP), gastrin-releasing peptide (GRP), [7] and prokineticin 2 [8], and the neurotransmitter GABA [9]. In addition, signals using the G-protein subunit G*_i/o_* [10] as well as gap junctions [11] have been implicated in the intra-SCN synchronization mechanism. The concept of mutual coupling, in which the neurotransmitter is released in a circadian fashion and feeds back on the clock, has been put forward by different authors [9,12–18]. Two recent studies analyzed the consequences of targeted disruption of genes coding for VIP or its receptor, VPAC_2_ [18,19]. In both cases, not only the synchrony between SCN neurons was lost, but, surprisingly, a majority of neurons also became arrhythmic. Similarly, inhibition of sodium channels by tetrodotoxin (TTX) desynchronizes and suppresses oscillatory activity in clock neurons [20]. In *Drosophila* also, electric disturbance of clock neurons can stop their free-running activity [21]. Activity at the neuronal membrane thus seems to play a role in maintenance of intracellular rhythms and coordination of neuronal clocks [22].

Here, we show that these results can be reproduced by a mathematical model of synchronization of coupled oscillators that are damped rather than self-sustained. Our model reproduces a number of experimental results well: (1) quick and robust synchronization under normal conditions; (2) loss of synchrony and rhythmicity in SCN slices after application of TTX, or in the absence of VIP signaling; and (3) entrainment by LD cycles. In addition, we show that if the number of oscillators is large enough and/or the connectivity between SCN neurons sufficiently strong, synchrony becomes a condition sine qua non for rhythmicity (i.e., the loss of coherent activity results in damped oscillations of individual neurons). Far from being coincidental, we suggest that synchrony-dependent rhythmicity in individual cells is a defining property of robustly synchronized systems like the SCN. Synchronization factors thus have a dual role in maintaining rhythmicity and synchronizing circadian oscillators.

## Results

### Structure of the Model

**Figure 1:**
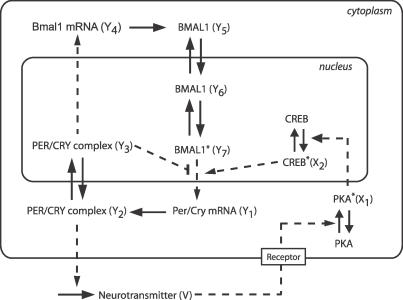
Scheme of the Single-Cell Circadian Oscillator, Including the Coupling Mechanism The intracellular oscillator consists of interlocked positive and negative transcriptional/translational feedback loops. In the negative feedback loop, *Per* and *Cry* genes (treated as a single variable) inhibit their own transcription by preventing BMAL1 from promoting *Per/Cry* transcription. In the positive feedback loop, the PER/CRY complex activates the transcription of their common transcriptional activator, *Bmal1* [23]. We assumed that the release of the neurotransmitter in the extracellular medium is activated by PER/CRY. In turn, the neurotransmitter activates a signaling cascade (involving PKA and CREB) that activates *Per/Cry* expression. In this schematic representation, solid arrows denote transport, translation steps, or phosphorylation/dephosphorylation reactions, while dashed arrows denote transcriptional regulations. The stars indicate the active (phosphorylated or complexed) form of the proteins. For a full reasoning of modeling assumptions see main text and [23].

**Figure 2:**
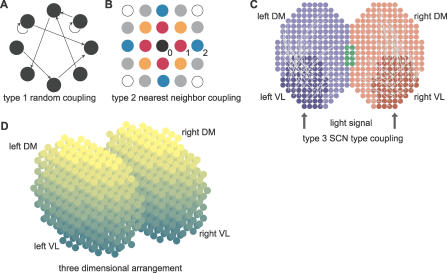
Organization of the Circadian Oscillator Networks (A) Random coupling (type 1). The probability that two oscillators are connected is independent of their positions. (B) Nearest-neighbor coupling (type 2). Oscillators are on a grid with a Euclidian distance *d*. Circle representing oscillators are color-coded for their distance from the central black oscillator. Black, red, orange, blue, gray, and white circles are at distances *d* = 0, 1,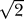 , 2,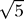 , and 2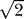 , respectively. Two oscillators are connected if their distance is less than a threshold *d_max_*. (C) SCN-like coupling (type 3). The SCN is divided in four regions, left and right VL regions (dark blue and red, respectively), and left and right DM regions (light blue and red, respectively; the green part is the intersection between left and right DM regions). Each dot represents an oscillator. Projections from the VL regions to their respective DM regions are indicated by light gray arcs. Projections from one cell to another are assigned randomly, with probability 0.5 for a DM cell to receive a projection. (D) Representation of a 3-D SCN. Each dot is a cell, and the color gradient indicates the VL–DM axis (dark cells are on the VL side and light cells are on the DM side, corresponding to the vertical axis in [C]). For type 3 coupling in a 3-D SCN, the regions are defined in the same way as in 2-D (C).

**Figure 3:**
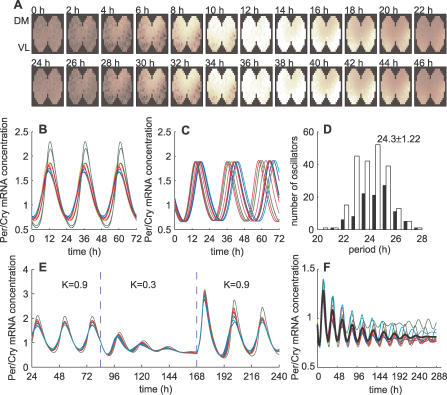
Synchronization of Damped Oscillators (A) Simulated evolution of *Per/Cry* mRNA expression over 48 h of 309 coupled neurons in a 2-D SCN slice with a type 2 coupling (nearest-neighbor coupling; *d_max_* = 3.5, *K* = 0.9). Dark brown corresponds to a low level of *Per/Cry* expression, while white corresponds to a high expression. (B) Time series of ten randomly chosen oscillators from the slice shown in (A). (C) Time series of ten randomly chosen uncoupled, free-running (*d_max_* = 0, *K* = 0.9) oscillators. (D) Distribution of cell-intrinsic periods (black, VL neurons; white, DM neurons; total number of cells, *n* = 309). (E) Time series of ten transiently weakly coupled oscillators (*d_max_* = 3.5, *K* = 0.3 between *t* = 84 and 168 h; *n* = 309). (F) Evolution of ten randomly picked oscillators simulating loss of VPAC_2_ receptor (*f* decreasing function of the distance, *d_max_* = 1, *K* = 0.45, *n* = 309). The decreasing *f* (see Equation 15) means that the signal is stronger for autocrine coupling. The thick black lines represent the average output. The resulting period is 24.7 h, and the average period of individual oscillators is 24.6 ± 1.2 h.

To simulate synchronization within the SCN, we constructed a network of coupled but damped molecular circadian oscillators. The model is built in two levels. First, on a single-cell level, we used a detailed molecular model to describe (1) the intracellular dynamics of clock genes and proteins, (2) the circadian neurotransmitter release by clock proteins, and (3) a simplified two-step signaling cascade leading to gene activation in response to neurotransmitter release (Figure 1). Second, on the “tissue” level, we placed the cells on a grid with the topology of a 2-D or 3-D SCN, and coupled them. We considered several coupling schemes mimicking different experimental conditions: (1) random sparse coupling (type 1, Figure 2A), (2) nearest-neighbor coupling (type 2, Figure 2B), and (3) SCN-like coupling combining nearest-neighbor and sparse coupling (type 3, Figure 2C).

#### Intracellular oscillator.

The molecular oscillator consists of a set of seven differential equations describing the time evolution of the key genes of the circadian clock, including *Per/Cry* and *Bmal1,* as proposed in a model by Becker-Weimann et al. [23] (Figure 1; Materials and Methods). In the present study we assume that in absence of synchronization signaling, intracellular oscillators are damped rather than self-sustained (see Introduction). Damped oscillators display a circadian rhythmic activity with gradually decreasing amplitude; without extracellular signals, rhythms vanish within a few days. To achieve damping, we slightly modified the parameter values of the original Becker-Weimann model (see Materials and Methods). In addition, to reflect experimental findings [4], we randomly assigned to each oscillator an individual, intrinsic period distributed around 24 h.

#### Coupling of oscillators.

The coupling between the molecular oscillators is assumed to be accomplished by a neurotransmitter released upon PER/CRY complex activity. The neurotransmitter triggers a signaling cascade that activates *Per/Cry* transcription both in the same cells as well as in coupled neighbors (Figure 1; Materials and Methods). The strength of the coupling signal depends on the average concentrations of neurotransmitter released by all coupled cells at a particular phase.

#### Topology of a population of oscillators.

To simulate SCN-like topology, the oscillators are disposed in a two-lobe–shaped 2-D or 3-D grid (Figure 2C and 2D). Each lobe represents one suprachiasmatic nucleus. The average random periods of oscillators and the connection between oscillators depend on their position within the grid. We simulated three types of coupling to reproduce various experimental conditions (see Materials and Methods). Type 1 is a random coupling with a nominal connectivity *c*_0_, which is the probability that two given oscillators are connected (Figure 2A). Such a coupling may be representative of conditions in neuron cultures dispersed at low or medium densities. Type 2 is a nearest-neighbor coupling, with connections only between oscillators that are separated by a distance smaller than a threshold *d*_max_. Type 2 coupling covers a broad class of locally coupled networks, as in high-density neuronal cultures (Figure 2B). Type 3 is aimed to reflect SCN-like coupling entrained by a LD cycle, where the grid is divided into four regions: each lobe is divided in a “ventrolateral” (VL) core and “dorsomedial” (DM) shell region (Figure 2C). Thus, we can simulate a different coupling type for each of the regions as well as coupling between the regions.

### Coupled Damped Oscillators Are Efficiently Synchronized

We studied the synchronization dynamics of coupled SCN neurons under four different conditions: high-density culture, low-density culture, presence of TTX, and loss of VIP/VPAC_2_ receptor.

First, we wanted to test how well coupled oscillators can synchronize under normal conditions that mimic wild-type SCN slices or neuronal cultures (high-density, no mutation). We simulated this by a nearest-neighbor coupling (type 2; Figure 3A, 3B, and Video S1) in a 2-D SCN slice geometry. In these simulations, we ignored spatial heterogeneity of the SCN except that we set the periods of the DM cells to be slightly shorter (4%) than those of the VL cells, consistent with experimental findings [24], and we distributed the periods around 24 h with a standard deviation of 5% [4,5]. As a readout for synchrony, we defined an order parameter *R* (Equation 18 in Materials and Methods). *R* is a normalized variance of the average *Per/Cry* mRNA concentrations in all cells, and varies between 0 (no oscillator synchronized) and 1 (all oscillators synchronized in phase). To describe the strength of the synchronization signal, we introduced a parameter *K* ≥ 0 that controls the overall coupling strength, and represents the sensitivity of cell to the neurotransmitter (for details, see Materials and Methods). With the coupling strength set to *K* = 0.9, the slice is well synchronized (*R* = 0.83), and the overall period is 24.4 h. The whole slice reached a stationary synchronized state less than 72 h after starting the oscillators from random initial conditions (Figure 3A and 3B; the first 72 h transients are not shown). Thus, the model reproduces well the high degree of synchrony seen in SCN slices.

The connectivity, defined as the average of the ratio between the number of connections and the maximal number of connections, is higher in 3-D (0.16) than in 2-D (0.10), as more neighbors are present within a given radius. Therefore, a complete SCN should synchronize even better than a 2-D slice. Indeed, simulations in a 3-D SCN geometry showed extremely well-synchronized cells (*R* = 0.97) with only a 2.5-h spread from the most advanced to the most delayed cells, compared with more than 4 h for a 2-D slice (Figure S2 and Video S2).

Second, having established that the model is well-synchronized under normal conditions, we wanted to know whether it could reproduce an SCN neuron culture dispersed at low density. To test this, we simulated a population of oscillators in which the neurotransmitter is only perceived by the cell that releases it (autocrine activation). Although individual oscillators are not self-sustained, simulations showed that isolated cells with autocrine activation become self-sustained oscillators, and oscillate with their intrinsic periods (Figure 3C and 3D). Thus, autocrine neurotransmitter activation seems to be sufficient to sustain oscillations in a dispersed cell culture. In addition, individual oscillators have an average intrinsic period of 24.3 ± 1.2 h that is very close to the period of the synchronized cells.

Third, we wanted to reproduce the loss of synchrony and rhythmicity in SCN slices after application of TTX. TTX blocks voltage-gated sodium channels and desynchronizes and suppresses oscillatory activity in clock neurons [20]. After removing TTX, the clock neurons resumed their oscillation and reestablished the same phase relationship as before TTX application. We simulated TTX experiments by using a weak coupling (*K* small) in a 2-D network with a nearest-neighbor coupling. We transiently decreased *K* from 0.9, as in normal conditions, to 0.3, and observed that all oscillators damped out. They quickly resumed their high-amplitude oscillations after restoration of full coupling (Figure 3E). Thus, the model reproduces the TTX experiments well.

Fourth, we simulated an SCN neuron culture in the absence of VIP signaling. Experimentally, in the absence of VPAC_2_, neurons show desynchronized and low-amplitude oscillations, or no oscillations [18]. In some cases, low-amplitude behavioral rhythmicity is retained [10,18,19,25], so we assumed that weak cell-to-cell interaction subsists [7,10] and decreases with the distance between cells. With such a severely impaired coupling, most of the oscillators rapidly damped out, while a few remained irregularly rhythmic for a longer time (Figure 3F). Simulations over a longer time of multiple slices confirmed that these are not self-sustained oscillations (i.e., single cells eventually become arrhythmic). The rhythmic average output is preserved in the first 144 h, with *R* = 0.78. Later, from 144 h to 288 h, *R* is considerably reduced (to 0.20), indicating a severe disruption of the synchrony after a few days.

### Single-Cell Rhythmicity and Synchrony Are Codependent

After having established that oscillators can be efficiently synchronized, and that weak coupling leads to loss of synchrony and rhythmicity in individual oscillators, we wanted to investigate whether coupled damped oscillators indeed only have two dynamic states: rhythmic and synchronized, or arrhythmic and desynchronized. To do this, we first recapitulated the simulations made in the previous subsection with only two coupled oscillators to explore all the dynamic states they can take. We then confirmed that in a large population, individual oscillators could not be rhythmic if their neighbors are not synchronized.

**Figure 4:**
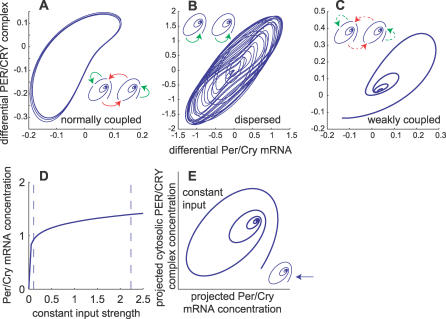
Single-Cell Rhythmicity Implies Synchrony (A–C) Phase space of a network of two oscillators showing possible dynamic behaviors. Each panel represents a condition that was simulated in the previous subsection. The inlets show what kind of coupling was considered (spirals, damped oscillators; solid arrows, normal coupling [*K* = 0.9]; dashed arrows, weak coupling [*K* = 0.3]; red and green arrows, paracrine and autocrine coupling, respectively). The axes show the differences of *Per/Cry* mRNA and PER/CRY complex concentrations between the two oscillators. This way, two oscillators can be represented in a 2-D space. (A) Normal, wild-type condition. The oscillators are normally coupled (autocrine and paracrine coupling), and the result is a regular, clock-like cycle denoting synchrony. (B) Dispersed condition. Oscillators with autocrine activation only are rhythmic, but quickly run out of phase. The result is an irregular cycle as phase differences are not constant. (C) Weak coupling. Oscillators with weak paracrine and autocrine coupling damp out to a steady state. (D) Stable steady state of *Per/Cry* mRNA under constant input. The minimal and maximal values of rhythmic input signals (variable *X*_2_) under normal coupling conditions are indicated by the dashed lines. (E) Phase space of a single oscillator with constant input. Because the intracellular oscillator is 7-D, we had to reduce the phase space from seven to two dimensions, and we chose a projection plane for which the trajectory was closest to a spiral.

We simulated possible dynamic outcomes of the coupling of two damped oscillators with random periods. We varied the coupling strength and the ability of the oscillators to sense autocrine or paracrine synchronization signals. First, if the oscillators sense strong autocrine and paracrine signals (*K* high enough, intercellular coupling), they synchronize (Figure 4A). Second, if the oscillators sense only autocrine signals (*K* high enough, no intercellular coupling), they oscillate, but do not synchronize (Figure 4B). Third, if the oscillators sense weak autocrine and paracrine signals (*K* small, intercellular coupling), their oscillations die out (Figure 4C). Despite many numeric simulations, we never encountered two normally coupled oscillators that are rhythmic but desynchronized. This indicates that in our model, rhythmicity is sufficient to induce synchronization, and vice versa.

A single oscillator in a large enough neighborhood of rhythmic but totally desynchronized cells would sense a constant average synchronization signal. In our model, the neurotransmitter activates the CREB protein in the signaling cascade. In simulations with constantly activated CREB protein (*X*_2_, Equation 9), oscillations stopped and a stable steady state was reached (Figure 4D). Any transient oscillatory activity damped out to that state (Figure 4E). Hence, a variable input is required for sustained rhythmicity of individual oscillators. In a large population of well-coupled cells, the variable input can only come, by definition, from synchronized neighbors. Noise is another source of variability that might affect synchrony. Two kinds of noise can be distinguished and can have different effects on the synchronization of oscillators. First, the noise can affect individual properties of oscillators (e.g., the successive periods of a given oscillator) or their coupling (e.g., the neurotransmitter released by each cell). Such a local noise impairs the synchrony as the strength of noise increases (Figure S3A–S3D). Alternatively, a spatially uniform extracellular noise could contribute to synchronize the cells (Figure S3E and S3F), even in the absence of synchronization signals, in much the same way that was described by Zhou and coworkers [26]. This result shows that synchrony is necessary for rhythmicity of single oscillators (i.e., single-cell oscillator rhythmicity and synchrony are codependent).

### Number of Oscillators and Connectivity Define Synchronization Properties

**Figure 5:**
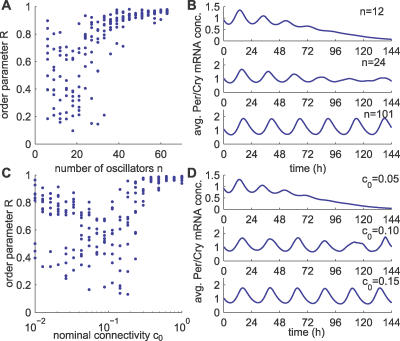
Effect of the Number of Oscillators as Well as the Connectivity on Synchronization (A) Synchronization properties of randomly coupled networks with respect to the number of neurons *n* (*c*_0_ = 0.10). Each dot represents the order parameter *R* for one realization of a random network and a simulation. Ten simulations were performed for each value of cell number *n*. The total length of the simulations was 312 h after starting with random initial conditions, and the order parameter was calculated over the last 240 h. (B) Three examples of average output for *n* = 12, 24, and 101. (C) Synchronization properties of randomly coupled networks with respect to the connectivity *c*_0_ (*n* = 12). Ten simulations were performed for each value of nominal connectivity *c*_0_. Other parameters as in (A). (D) Three examples of average *Per/Cry* mRNA concentration for *c*_0_ = 0.05, 0.10, and 0.15.

So far, we have looked at a large number of oscillators and found that robust synchronization is achieved when oscillators are appropriately coupled. To analyze the influence of the number of oscillators as well as the connectivity on synchronization dynamics, we used a uniform, random coupling (type 1), and we varied either the number of oscillators or the nominal connectivity (Figure 5) and measured the *R* values.

First, we considered the synchronization of ensembles consisting of six to 63 oscillators, with a nominal connectivity *c*_0_ = 0.1. For the larger ensembles, strong synchronization was consistently achieved (*R* > 0.8 for *n* > 40). For smaller ensembles, the order parameter *R* shows a high variance, ranging from 0.15 to almost 1 for *n* = 27. High variability of *R* values denotes poor, nonrobust, network-dependent synchronization (Figure 5A). Representative average outputs for small cell numbers are damped or irregular compared with larger networks (Figure 5B, two top panels versus bottom panel).

Second, we tested the influence of the connectivity on synchronization properties (*c*_0_ ranging from 0.005 to 1 with a fixed number of cells *n* = 12). For dense networks (*c*_0_ ≥ 0.5), synchronization was consistently excellent (*R* > 0.9). Sparsely connected networks (*c*_0_ < 0.5) result in highly variable *R* values, as for small numbers of oscillators. For small *c*_0_ values (<0.1), we observed better synchronization, perhaps because usually only one synchronized cluster forms (Figure 5C). Sparsely coupled networks show dynamics similar to small population networks, as the connectivity is varied (Figure 5D). These results show that in random networks, both a sufficient number of oscillators and connectivity contribute to strong and robust synchronization. In weakly coupled networks, in addition to a loss of rhythmicity, *Per/Cry* mRNA concentration decreases exponentially (Figure 5B and 5D, top panels), consistent with the relative “dark” cells observed in VPAC_2_ receptor–deficient luciferase reporter mice [18].

For large numbers of SCN neurons, as in vivo (hundreds to thousands), we found that synchrony is achieved even for very small connectivity values. Therefore, a larger number of neurons in the network ensures that even a great reduction in connectivity will not impair synchrony.

### Slow Oscillators Have a Higher Impact on the Period

Mutations in clock genes that modify the free-running period of the SCN provide a way to test the synchronization properties of neurons with different periods. Hamsters homozygous for the *tau* mutation have free-running periods of about 20 h compared with 24 h in wild-type hamsters [27]. The free-running periods in mutant and wild-type animals are determined by the average of periods of dispersed individual clock cells [28,29]. In a recent experiment by the Herzog lab, when dispersed SCN neurons of *tau* mutants and wild-type hamsters were mixed in cell cultures, the resulting period of the total population turned out to be longer than the average period of the two unmixed populations, which would have been the naive prediction (S. Aton and E. Herzog, personal communication).

**Figure 6:**
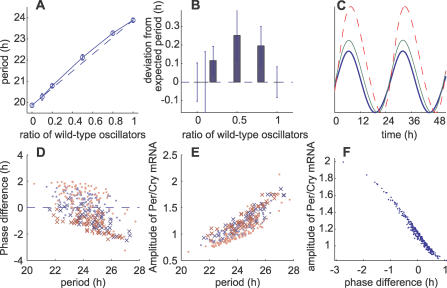
Effect of the Intrinsic Period on Amplitude and Phase (A) Resulting periods of two mixed populations of oscillators, one with a 24 h period and one with 20 h period (type 1 coupling; *c*_0_ = 0.1). The period was calculated with proportions of 24 h period cells of 0.0, 0.1, 0.2, 0.5, 0.8, and 1.0, and results of five runs for each proportion were averaged (total *n* = 100, [open circles] average period). The dashed line represents the average of the individual oscillators' periods. (B) Deviation of the resulting population periods shown in (A) from the average of the individual oscillator periods (the error bars are the standard deviations). (C) Three coupled oscillators with different intrinsic periods showing their phase and amplitude relationships: the short period oscillator (thick blue line) is phase-advanced and low amplitude compared with the average period oscillator (green line) and the long period oscillator (dashed red line). (D) Phase difference from the average *Per/Cry* mRNA concentration with respect to the intrinsic periods, from the simulations shown in Figure 3A and 3B (dark blue ×, left VL neurons; light blue, left DM neurons; dark red ×, right VL neurons; light red, right DM neurons). A positive phase difference means phase-advanced compared with the phase of the population. (E) Amplitude with respect to the intrinsic period from the simulations shown in Figure 3A and 3B (color code as in [D]). (F) Amplitude phase relationship for type 1 coupling (*K* = 0.9, *c*_0_ = 0.1, number of oscillators *n* = 100).

With this experimental observation in mind, we tested whether our model might be able to explain this nonintuitive result. We randomly mixed and connected a 24 h–period population with a 20 h–period population (total, *n* = 100; standard deviations of the periods, 0.1 h) in various ratios. The resulting period of the synchronized population was compared with the period that would have been expected from averaging the individual oscillator periods. For ratios between 0.2 and 0.8 (i.e., 20% and 80% wild-type cells), the resulting population period was systematically longer than expected (five runs per ratio, *R* > 0.8; Figure 6A and 6B). Thus, in mixed population, slow oscillators seem to have a higher impact on the period than the faster ones.

In a synchronized SCN neuron slice culture or a high-density SCN neuron dispersal culture, the intrinsic periods of the neurons (i.e., of the noncoupled neurons) cannot be determined experimentally, only the phases and amplitudes. Thus, we used our model to relate these two measures to the intrinsic periods of the neurons. To this end, we extracted the intrinsic periods of the neurons by uncoupling them and calculating their free-running periods. We saw that, when coupled, oscillators with short periods are phase-advanced, and oscillators with long periods are phase-delayed compared with the phase of the average output of the population (Figure 6C and 6D), consistent with the observation that the DM region is advanced with respect to the VL region [20]. We also saw that the amplitudes of oscillators are higher when their periods are longer (Figure 6E). Consequently, oscillators with high amplitudes are phase-delayed, and oscillators with low amplitudes are phase-advanced (Figure 6F). These findings explain why synchronized oscillators have a period longer than expected (i.e., because high-amplitude oscillators contribute more to the population than those with small amplitude).

### The Light-Entrained Core Oscillators Can Entrain the Shell Oscillators

An important property of the circadian clock is its capability to be entrained by daily LD cycles. The light signal is conveyed from the retina to the SCN via the retino-hypothalamic tract [30]. Retino-hypothalamic cells release glutamate and PACAP, which activate *Per* gene expression in the target VL cells [31], which then relay the light signal to the DM cells [17]. After a phase-shift in the LD cycle, the light-responsive VL neurons re-entrain rapidly (∼2 d) to the new schedule, while the DM neurons take much longer to readjust—up to 13 d after a 6-h advance in the LD cycle [32].

**Figure 7:**
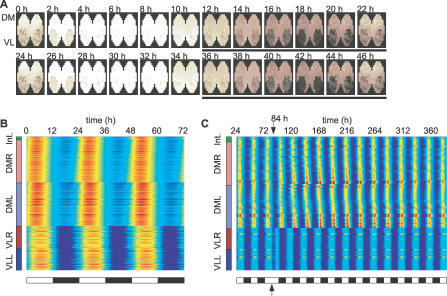
Simulation of Entrainment of a 2-D SCN Slice by a 12:12 LD Cycle (A) Simulation of the evolution of *Per/Cry* mRNA over 48 h of 309 coupled cells (VL, *n* = 102; DM, *n* = 207) in a 2-D SCN slice with a type 3 coupling (*d_max_* = 3.5, 50% of neuronal projections, 4% average period gradient, *K* = 1.0, *L*_0_ = 0.22). The black bars indicate a dark phase (color code as in Figure 3A). Individual oscillators have an average period of 23.7 ± 1.2 h. Initial conditions were chosen randomly. The first 72 h of transient were discarded, and the time from 72 h to 144 h was retained. (B,C) Raster plot of *Per/Cry* mRNA activity in oscillators, organized according to their regions (from bottom up: VLL, left VL region; VLR, right VL region; DML, left DM region; DMR, right DM region; and Int, intersection between left and right DM regions). The concentration of *Per/Cry* mRNA for each oscillator is represented by colors (blue, low concentration; red, high concentration). (B) 12:12 LD cycle. (C) 12:12 LD cycle with a 12 h phase shift at *t* = 84 h.

To test whether our model is able to entrain to LD cycles and to analyze entrainment dynamics, we simulated a 12 h–12 h LD cycle in a 2-D SCN with a type 3 coupling by imposing a periodic forcing on the expression of *Per/Cry* gene in the VL cells. Through neuronal projections, VL cells entrained the DM cells (Figure 7A and 7B, and Video S3). Starting from completely desynchronized cells, high synchrony (*R* = 0.92) and phase-locking to the LD cycle (with a 24-h period) are reached very fast, within 72 h. The phases of DM cells were slightly more advanced than those of the light-inducible VL cells (unpublished data), as observed experimentally [20]. After a 12-h phase-shift in the LD cycle, VL cells resumed their phase quickly (after 2 d), while DM cells took more than 10 d to resynchronize to the LD cycle (Figure 7C). These results are in agreement with experimental findings [32], and show that entrainment by a LD cycle is efficient even if only a fraction of the cells can respond to the light signal (102 out of 309), but also that the light-insensitive cells take a longer time to adjust their phase.

## Discussion

### A Model for the SCN Circadian System

Recent technological advances made it possible to measure the oscillation dynamics of single neurons within a SCN tissue at a high resolution, thus providing experimental data to construct and support more realistic SCN models [3,19]. Several papers proposed models for the molecular mechanism underlying circadian oscillations at the single-cell level [23,33–35], but without considering intercellular communication. Other studies considered intercellular coupling mechanisms between generic oscillators without taking into account the influence of the rhythmicity of the intercellular coupling on the oscillators themselves. In two models, the intracellular oscillator is a van der Pol oscillator, which is a generic two-variable system displaying strong self-sustained oscillations. The models differ in the way cells are coupled: Kunz and Achermann [36] showed how uniformly locally coupled networks can robustly synchronize, while Antle and coworkers [37,38] proposed that a subset of gate cells provide daily inputs to rhythmic oscillators. Rougemont and Naef [39] used more abstract Kuramoto oscillators, in which only the phase (not amplitude) is described, with periods and phases randomly varying in time to characterize the source of phase dispersion. The first attempt to describe synchronization of circadian oscillators that are based on realistic genetic network was by Ueda et al. [40], who showed that synchronization factors confer noise resistance to circadian rhythms in populations of oscillators. Roenneberg and Merrow [41] proposed the concept of zeitnehmer, where the cellular circadian oscillator feeds back on the input pathways of the zeitgebers, blurring the distinction between intra- and extracellular components. Here, we present a molecular model for the SCN circadian system that combines intracellular and extracellular dynamics at the single-cell level.

So far, all published models assumed that individual oscillators are self-sustained. Recent experimental observations challenge that assumption. SCN slices treated with TTX, an inhibitor of sodium channels, lose both synchronization and rhythmicity [20]. In VIP and VPAC_2_ receptor–deficient high-density neuron dispersals, about 70% of the neurons are no longer rhythmic [19]. Similarly, in the slices from mice lacking the VPAC_2_ receptor, only a minority of neurons from the dorsal shell is rhythmic, and shows poorly organized and low-amplitude circadian gene expression [18]. These results suggest that synchronization factors are not only required for synchrony, but also for rhythmicity of individual cells. Therefore, in the present model, we considered a population of oscillators that are damped in the absence of synchronization signals.

We built a heterogeneous network of coupled damped circadian oscillators. On a single-cell level, we used a molecular model of the circadian clock [23], neurotransmitter release by clock proteins, and signaling cascade that leads to clock gene activation. We obtained a damped intracellular oscillator by reducing the steepness of the *Per/Cry* promoter feedback loop (Hill coefficient). The Hill coefficient represents the cooperative character of the transcriptional inhibition process. A lower Hill coefficient leads to a more gradual inhibition of the promoter, whereas a high Hill coefficient results more in a switch-like process. On a population level, we placed the cells on a grid with a flexible topology of a 2-D or 3-D SCN, and coupled them. The phenotypes of the neurons (period, amplitude, sustained or damped activity, neuropeptide release and receptor expression, connectivity, etc.) were specified according to their position in the grid.

### Comparison with Experimental Results and Predictions

We verified that our model reproduces well-known behaviors of SCN. In high-density networks, the modeled coupled oscillators are rhythmic and well synchronized in absence of external cues (Figure 3A and 3B). We simulated TTX treatment of neurons [20] by lowering the coupling strength and showed that rhythmic activity in single oscillators disappeared and resumed quickly after the full coupling was restored (Figure 3E). Then, we simulated loss of VIP and VPAC_2_ receptor [13,18,19] by lowering the coupling strength and reducing the range of connectivity, and showed that oscillators were slowly desynchronized and damped (Figure 3F). We assumed the presence of a short-range coupling because in the absence of the VPAC_2_ receptor, mice express multiple circadian periods over more than 80 d when kept in constant darkness [19], suggesting the existence of isolated islands of synchronized, locally coupled SCN neurons. We also verified that a periodically entrained subset of neurons (the VL core) could entrain the rest of the neurons (the DM shell) to a 24-h period (Figure 7A and 7B). After simulating a 12-h phase-shift in the LD cycle, the light-inducible VL region reset its phase much faster than the DM region (2 d versus 10 d; Figure 7C).

Damped oscillators in a large coupled population can adopt two and only two dynamic behaviors, depending on the coupling: (1) damping if uncoupled or weakly coupled, or (2) synchrony if normally coupled (Figure 4). A direct consequence is that coupled cells cannot run out of phase and still oscillate (individual cells dispersed at low density are viewed as many independent synchronized systems). The coupling of damped oscillators produces a circadian pacemaker that is robustly synchronized: provided they are rhythmic, neurons will synchronize. If some neurons lose synchrony, they will damp out, leaving the rest of the SCN unperturbed.

We showed that to achieve robust synchronization, the number of neurons and the connectivity matter (Figure 5). In neuron dispersals, coherent rhythmic output is density-dependent [4,5,19]. In addition, Yamaguchi et al. [20] reported that the upper dorsal region of a SCN slice lost its rhythmicity when cut out from the ventral region, perhaps because of the small size of the separated region—25 neurons were measured in the cut piece. In VPAC_2_ receptor–deficient or VIP-deficient mice, simultaneous multiple free-running periods in behavior could result from parallel, synchronized clusters in loosely connected networks.

Ohta et al. [42] reported that after 3–5 mo, 10% of the mice kept in constant light showed arrhythmicity. They showed that the arrhythmicity is due to desynchronization between rhythmic SCN neurons. The only way our model could reproduce these results is by decreasing paracrine coupling without interfering with autocrine coupling. But at present, there is no evidence that constant light could induce such a selective disruption.

In a driven harmonic oscillator like a pendulum, the highest amplitude is achieved when the driving period and the intrinsic period coincide [43]. Unexpectedly, in our model, we obtained a monotonic curve in which the amplitudes increase with the periods, possibly because of the interaction between *Per/Cry* gene activation by the synchronization signal and BMAL1 protein (Figure 6E). As a result, slow oscillators have a higher impact on the period in a mixed population, qualitatively reproducing the results from a mixed-genotype experiment (E. Herzog and S. Aton, personal communication). This contrasts with mutually coupled threshold-activated oscillators, where the fastest elements set the period [6]. In homogenous cell cultures, the difference between the free-running period and the average period of individual neurons is smaller than what is statistically detectable [29]. Our simulations also showed no statistical difference between average and synchronized periods (simulations from Figure 3A–3D, two-sided *t-*test, *p* = 0.20).

Based on our results, we propose three experimentally testable predictions. (1) Oscillations in a majority of VPAC_2_ receptor–negative neurons dispersed at low density should rapidly damp out after induction by serum shock. If validated, this would confirm that loss of rhythmicity in a VPAC_2_ receptor–negative SCN slice is not due to unexpected cell–cell interaction. To test that neurons need periodic synchronization signals to be rhythmic, one could treat VIP-deficient neurons with constant high levels of VPAC_2_ agonist. We predict that arrhythmic neurons will stay arrhythmic. (2) A low number of neurons or a low connectivity should result in desynchrony. Medium-density neuron cultures with a small number of neurons should display variability in their synchrony levels (including nonoscillatory neurons as defined by the order parameter *R*). Increasing the density or the number of neurons would reduce the variability of synchronization levels and increase the average synchrony. Knife cuts in SCN slices to isolate different numbers of neurons could be a way to test size dependency. We predict that pieces that contain fewer than 40 neurons will display large variations in synchronization levels. In mice heterozygous in the gene coding for the VPAC_2_ receptor, SCN neurons seem to have synchronization properties similar to those in wild-type mice [18]. However, if connectivity is subtly altered in heterozygous mice, a prediction is that asynchrony will occur in larger-cut SCN pieces than for wild-type mice. (3) In high-density dispersal cultures, normalized amplitudes [44] of oscillations should be negatively correlated with the phases as in Figure 6F, provided there is a small variation in the natural amplitudes of isolated neurons. This would be a way to estimate the free-running periods of individual neurons without the need to disperse them.

### Synchronization Mechanism

The synchronization of damped oscillators is independent from the particular intracellular model used. Systems with a Goodwin-type model as used in [45], the Leloup-Goldbeter model [33], and other simple negative feedback oscillators have similar synchronization properties (Figure S4–S6). Numeric exploration of such models suggests that positive feedback loops facilitate, but are not necessary for, efficient synchronization (unpublished data). The variability of behavioral periods in *Rev-Erb*α knockout mice, in which the positive loop is dysfunctional, could reflect that feature [46]. One could test whether synchronization properties of SCN neurons are altered in these mice by analyzing SCN neurons from *Rev-Erb*α^−/−^Per2:luc double-transgenic mice. We predict that *Rev-Erb*α knockout mice will have lower amplitude and more spread-out synchronized SCN neurons. Li and coworkers [47] introduced “transient resetting” as a possible synchronization mechanism, in which uncoupled oscillators are synchronized by a force (which may be noise) that transiently moves them to a region where they have a stable steady state. In our model, the driving force was generated autonomously by the coupled oscillators. To our knowledge, it is the first time that synchronization-induced rhythmicity is described in a biological system. Damped but uncoupled oscillators have been considered before in a model of interaction between a clock and a zeitgeber input pathway [41]. Temporal and spatial rhythms can occur when identical stable systems are diffusively coupled together, giving rise to well-studied Turing instabilities [48,49]. Oscillations can also emerge from electrical coupling between nonoscillating cells [50,51]. Nonetheless, two features distinguish our work from that mentioned above. (1) In our system, temporal instabilities do not arise from spatial heterogeneity or local coupling because synchrony also holds in case of all-to-all coupling of identical oscillators. (2) Oscillators are directly coupled, instead of being diffusively coupled [52]. Direct coupling means that even under perfect synchrony, the coupling term is nonzero, unlike in the diffusive coupling case.

The question of how a coherent and robust circadian output is generated from a heterogeneous network of 20,000 oscillators in the SCN has led to many surprising results [2,3,18–20], bringing a better understanding of the interaction between the single-cell clock and its organization. To understand the orchestration of timekeeping in the SCN, intracellular circadian modules cannot be isolated from their intercellular communication components.

## Materials and Methods

### Single-cell oscillator model.

The intracellular oscillator is a seven-variable model representing clock genes' mRNA and proteins [23]. It consists of interlocked transcriptional/translational feedback loops and reflects the essential features of the mammalian circadian oscillator (Figure 1). The circadian release of a neuropeptide mediates intercellular coupling of circadian cells in the SCN [9,12–18]. Here, we assumed that the release of the neuropeptide is induced by the cytosolic PER/CRY protein complex. The neuropeptide activates a two-step cascade in connected cells that leads to *Per/Cry* mRNA transcription. The assumption that the PER/CRY complex induces neuropeptide release was made to ensure that the transmitter is released quickly after *Per/Cry* gene activation. The cascade is schematized by PKA and CREB activation. With the neurotransmitter and the two-step cascade, the complete single-cell system has ten variables.

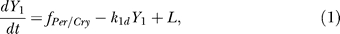


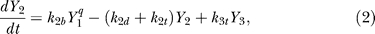


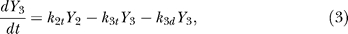


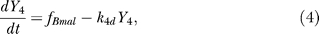


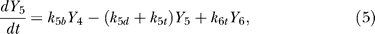


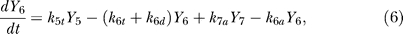


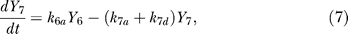


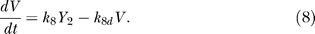



The nonlinear transcription functions are

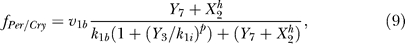


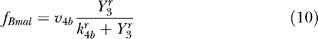



The coupling term *Q* induces a signaling cascade leading to activation of *Per/Cry* promoter, and is proportional to the local mean field *F*.

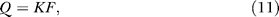


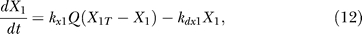


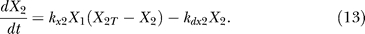



The variables represent the following species: *Y*_1_, *Per/Cry* mRNA; *Y*_2_, PER/CRY cytosolic complex; *Y*_3_, nuclear PER/CRY complex; *Y*_4_, *Bmal1* mRNA; *Y*_5_, cytosolic BMAL1; *Y*_6_, nuclear BMAL1; *Y*_7_, transcriptionally active BMAL1*; *V*, neurotransmitter; *X*_1_, PKA; and *X*_2_, CREB. *F* is the local mean field as defined in Equation 17, and *K* is a scalar determining the coupling strength. Equations for *X* and *Y* are replicated *n* times, where *n* is the number of cellular oscillators (we omitted the indices *i* for readability). The entry *Q_i_* corresponds to the coupling term in cell *i*, *i* = 1,...,*n*. Furthermore, each system is scaled by a factor *e_i_* to generate a distribution of periods. For *e_i_*, we generated a sample *g_i_* drawn from a Gaussian distribution centered at 1 with a standard deviation of 0.05. Additional heterogeneity was added in the form of a vector *u_i_* that defines a linear or radial gradient of periods according to the position of the cells in the SCN. The scaling factor *e_i_* is defined as

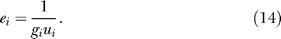



This produces a distribution of periods between 20 and 28 h.

The parameter values for the model are *v*_1*b*_ = 9.0, *k*_1*b*_ = 1.0, *k*_1*i*_ = 0.56, *p* = 3, *h* = 2, *k*_1*d*_ = 0.18, *k*_2*b*_ = 0.3, *q* = 2, *k*_2*d*_ = 0.1, *k*_2*t*_ = 0.36, *k*_3*t*_ = 0.02, *k*_3*d*_ = 0.18, *v*_4*b*_ = 1.0, *k*_4*b*_ = 2.16, *r* = 3, *k*_4*d*_ = 1.1, *k*_5*b*_ = 0.24, *k*_5*d*_ = 0.09, *k*_5*t*_ = 0.45, *k*_6*t*_ = 0.06, *k*_6*d*_ = 0.18, *k*_6*a*_ = 0.09, *k*_7*a*_ = 0.003, *k*_7*d*_ = 0.13, *k*_8_ = 1.0, *k*_8*d*_ = 4.0, *K* = 1.0, *k_x_*_1_ = 3.0, *X*_1*T*_ = 15.0, *k_dx_*_1_ = 4.0, *k_x_*_2_ = 0.25, *X*_2*T*_ = 15.0, *k_dx_*_2_ = 10.0, and *L*_0_ = 0.22. Rates *k* are in h^−1^ except *k*_2*b*_ (h^−1^nM^−(*q*−1)^), *k*_1*b*_ (nM), *k*_1*i*_ (nM), and *v*_4*b*_ (nM), *k_x_*_1_ and *k_x_*_2_ (h^−1^nM^−1^), *v* and *L*_0_ (nM h^−1^), and *X_T_* (nM). Parameters were minimally adapted from the original model [23] to satisfy the following conditions: individual oscillators must be damped, and the coupled system must synchronize with a circadian period. Specifically, we reduced the Hill coefficient *p* from 8 to 3. A small Hill coefficient makes the periods longer, so we compensated the periods by increasing the degradation rates.

### Coupling of neuronal circadian oscillators.

Neuropeptide release and action in the intercellular medium are fast compared with the 24-h period of the neurons, allowing diffusion and transport delays between connected cells to be neglected. For a given neuron, we defined a local mean field as the average concentration of neurotransmitter released by the neighboring (connected) cells. This type of coupling is termed direct, as opposed to a diffusive coupling [52].

We considered two different shapes for the SCN. (1) The whole SCN is defined on a 3-D discrete cubic grid *G,* of size *s,* where each nonempty node represents a neuron (Figure 2D). Each neuron (node) is assigned a number from 1 to *n,* and empty nodes have a value 0. Functional or physical regions of the SCN are defined by subgrid *E* of *G* by retaining as nonempty only the nodes belonging to the desired region. This way, various overlapping regions can be defined. (2) An SCN slice is defined on a 2-D square grid in a manner analog to the whole SCN (Figure 2C). Regions of the slice are constructed the same way as in 3-D. We used a Euclidian distance *d*(*i*,*j*) to measure the distance between neurons *i* and *j*, with *d* = 1 for adjacent neurons (Figure 2B).

A coupling matrix *M,* which depends on the geometry of the SCN and a connectivity map *C,* describes the connections between cells. A neuron *i,* belonging to a population of size *n,* receives an input from neuron *j* if *C_i,j_* is 1. We considered three different types of coupling for *C* (Figure 2A–2C and Figure S1).

*Random coupling (type 1): C_i,j_* = 1 with probability *c*_0_ (the nominal connectivity) (Figures 2A and S1A). All-to-all coupling is a particular case of sparse coupling when *c*_0_ = 1.

*Nearest-neighbor coupling (type 2): C_i,j_* = 1 if *d_i,j_* < *d_max_* and neuron *i* is downstream of neuron *j* (Figures 2B and S1B).

*SCN-like coupling (type 3):* we divided the SCN into four regions: left and right VL, and left and right DM regions. The VL part (the ventral core) corresponds to the light-inducible cells and has many projections into the DM region [3,53]. Neurons within the VL part are not coupled and are not spontaneously rhythmic [3,18]. The DM region (the dorsal shell) receives the input from the VL neuronal projections. We used a uniform type 2 coupling (nearest-neighbor) to couple DM cells (Figures 2C and S1C). We used this coupling type in conjunction with an LD cycle.

In addition to the connectivity, a function *f*(*d*) determines the relative coupling strength between neurons separated by a distance *d*. Because of the mean field assumption, the effect of all neurons upstream of neuron *i* is averaged, so the coupling matrix *M* ∈ R*^n^* ^×*n*^ is

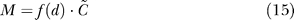
where the dot (·) denotes the element-wise matrix product and *C~* is normalized so that the sum of each line is 1,

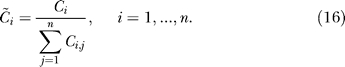



The matrix *C* is normalized as a result of the local mean field assumption: the input to one cell is the average of the signal coming from upstream neurons. The fraction of nonzero entry of *C* is the connectivity, a scalar denoted by *c*. The input at each neuron in the SCN is described by the mean field vector *F* ∈ R*^n^*,

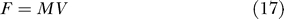
where *V* is the transmitter concentration (Equation 8). To measure synchrony, we used an order parameter *R* [54] defined as

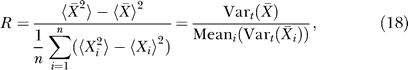
where 〈...〉 denotes the average over time, and 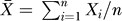 is the average of the variable of interest among oscillators. For comparison with bioluminescence recordings, we chose the variable of interest to be *Per/Cry* mRNA concentration, *Y*_1_ in Equation 1 (half-lives of the reporters are short enough for the reporter itself to be neglected [20]).

We simulated light entrainment by a clipped sine wave,

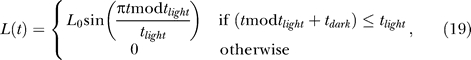
(0 < *L*(*t*) ≤ *L*_0_ and *L*(*t*) = 0 alternating every *t_light_* and *t_dark_* h).

Unless specified, initial conditions for each simulation were randomly chosen, with each variable taking a value between 0 and 2 times the average value of the variable when the system is synchronized. Simulations and analysis were performed in the Matlab 6.5 environment (The MathWorks, http://www.mathworks.com). The ordinary differential equations were simulated with the medium-order adaptive step solver ode45. The codes are available on request.

## Supporting Information

Figure S1 Coupling Matrices *C* for the Three Types of Coupling In the “spy” matrix representation, the presence of a dot at position (*i*,*j*) denotes a directional coupling from cell *j* to cell *i* (*C_i,j_* = 1), and a blank space means that there is no coupling (*C_i,j_* = 0).
(A) Matrix *C* for the random coupling type (type 1).
(B) Matrix *C* for the nearest-neighbor coupling type (type 2). The matrix is symmetrical; hence, all coupling is bidirectional.
(C) Matrix *C* for the SCN coupling type (type 3). The red and blue shades represent DM intercellular coupling, and the black dots represent projections from the VL to the DM regions.
(148 KB PDF)

Figure S2 Raster Plot of 625 Oscillators with Nearest-Neighbor (Type 2) Coupling in a 3-D SCN Geometry SCN geometry is organized according to their regions (from bottom up, VLL: left VL region, VLR: right VL region, DML: left DM region, DMR: right DM region, and Int: intersection between left and right DM regions). The concentration of *Per/Cry* mRNA for each oscillators is represented by colors (blue, low concentration; red, high concentration). All other parameters are as in Figure 3A and 3B.
(1.9 MB PDF)

Figure S3 Synchronization of Ten All-to-All Coupled Oscillators with Noisy Coupling and Noisy Intrinsic Periods We used a multiplicative noise described by stationary Gaussian process (Ornstein-Uhlenbeck process; see Text S1) with noise strength *S*.
(A,B) Synchronization-coupled oscillators with noisy transmitter release and noisy intrinsic periods (*S* = 0.03, *K* = 0.9). Simulations were made over 2,400 h ([A]; gray lines, individual oscillators; blue line, average of the ten oscillators). Synchronization was good (*R* = 0.89) despite the variations in amplitudes and periods (B).
(C) Coupling improves temporal precision. Successive periods of the average output of synchronized oscillators (black bars, label P) show a better precision than individual oscillators in the synchronized state (gray bars, label C) and uncoupled oscillators (white bars, label U). The standard deviations were 0.5 h, 0.6 h, and 1.9 h, respectively, for data from simulations shown in (A) and (B).
(D) Effect of noise strength *S* on the order parameter *R* and the standard deviation of periods of the average output of coupled oscillators. Synchronization was good (*R* > 0.8; blue lines) and standard deviation limited (<2 h; red dashed lines) for *S* below 0.05. For *S* larger than 0.05, synchronization was still achieved, but the period became unreliable.
(E,F) Global noise can enhance synchrony. In the presence of strong noise (*S* = 0.2), weakly coupled oscillators (*K* = 0.4) maintained rhythmicity and synchrony (*R* = 0.94) (E). For weaker noise (*S* = 0.05), the same weakly coupled oscillators did not maintain rhythmicity (F).
(273 KB PDF)

Figure S4 Synchronization of 309 Goodwin-Type Damped Oscillators with Nearest-Neighbor (Type 2) Coupling The simulation is divided in four parts to show synchrony, damping, restoration of synchrony, and damping under constant signal. First, in constant darkness (DD) with normal coupling (*K* = 1.5), oscillators are well-synchronized. Second, in DD without coupling (*K* = 0), oscillators lose synchrony and rhythmicity. Third, in DD, normal coupling (*K* = 1.5) restores synchrony and rhythmicity. Fourth, in constant light (LL) without coupling, oscillators lose rhythmicity, showing that they are damped in the absence of a variable signal.
(20 KB PDF)

Figure S5 Synchronization of 100 One-Variable Negative Feedback Loop Oscillators with Delay (All-to-All Coupling) The simulation is divided into four parts to show synchrony, damping, restoration of synchrony, and damping under constant signal. First, in DD with normal coupling (*K* = 0.1), oscillators are well-synchronized. Second, in DD without coupling (*K* = 0), oscillators lose synchrony and rhythmicity. Third, in DD, normal coupling (*K* = 0.1) restores synchrony and rhythmicity. Fourth, in LL without coupling, oscillators lose rhythmicity, showing that they are damped in the absence of a variable signal.
(21 KB PDF)

Figure S6 Synchronization of 40 Leloup-Goldbeter Oscillators with All-to-All Coupling The simulation is divided into four parts to show synchrony, damping, restoration of synchrony, and damping under constant signal. First, in DD with normal coupling (*K* = 1), oscillators are well-synchronized. Second, in DD with weak coupling (*K* = 0.6), oscillators lose synchrony and rhythmicity. Third, in DD, normal coupling (*K* = 1) restores synchrony and rhythmicity. We applied a light pulse to shorten the transients. Fourth, in LL without coupling, oscillators lose rhythmicity, showing that they are damped in the absence of a variable signal.
(128 KB PDF)

Text S1 Supplementary Materials and Methods (50 KB PDF)

Video S1 2-D Slice with Nearest-Neighbor (Type 2) Coupling over 72 h (*d_max_* = 3.5, *K* = 0.9) The time series are also shown in Figure 3A and 3B. Each dot is an oscillator. The concentration of *Per/Cry* mRNA for each oscillator is represented by colors (blue, low concentration, red, high concentration) and size.
(2.0 MB AVI)

Video S2 3-D SCN with Nearest-Neighbor (Type 2) Coupling over 72 h (*d_max_* = 3.5, *K* = 0.9) Except for the 3-D geometry, all parameters are as in Figure 3. Each dot is an oscillator. The concentration of *Per/Cry* mRNA for each oscillator is represented by colors (blue, low concentration; red, high concentration) and size. The SCN is slowly rotating to show the 3-D structure.
(1.5 MB AVI)

Video S3 2-D Slice with Type 3 Coupling over 72 h under a 12:12 LD Cycle Total *n* = 309, VL regions *n* = 102, DM regions *n* = 207 *d_max_* = 3.5, 50% of neuronal projections, 4% average period gradient, *K* = 1.0, *L*_0_ = 0.22. The time series are also shown in Figure 7. Each dot is an oscillator. The concentration of *Per/Cry* mRNA for each oscillator is represented by color (blue, low concentration; red, high concentration) and size.
(2.0 MB AVI)

## Abbreviations

DM: dorsomedial
LD: light–dark
SCN: suprachiasmatic nuclei
TTX: tetrodotoxin
VIP: vasoactive intestinal polypeptide
VL: ventrolateral
