# Supplementary material for: Synchronization-Induced Rhythmicity of Circadian Oscillators in the Suprachiasmatic Nucleus
Source: PLoS Comput Biol. 2007 Apr 13;3(4):e68. doi: 10.1371/journal.pcbi.0030068 (PMC1851983; doi:10.1371/journal.pcbi.0030068)
Supplement: Supplementary file 1 [file pcbi.0030068.sd001.pdf]

Text S1

Goodwin-type 3-variable model

The Goodwin-type model consists of three differential equations with a negative feedback loop and Michaelian degradation terms. When the Hill coefficient is sufficiently high ($h > 4$), the system exhibits self-sustained oscillations in absence of any external signals (1). For lower values of h, the system has only a stable steady state.

$$\frac{\mathrm{d}X}{\mathrm{d}t} = v_1 \frac{K_1^h}{K_1^n + Z^h} - v_2 \frac{X}{K_2 + X} + v_c \frac{Q}{1+Q} + L,$$
$$\frac{\mathrm{d}Y}{\mathrm{d}t} = k_3 X - v_4 \frac{Y}{K_4 + Y},$$
$$\frac{\mathrm{d}Z}{\mathrm{d}t} = k_5 Y - v_6 \frac{Z}{K_6 + Z},$$
$$\frac{\mathrm{d}V}{\mathrm{d}t} = k_7 X - v_8 \frac{V}{K_8 + V}.$$

The parameter values are v_1=1.2, K_1=0.6, h=3, v_2=0.35, K_2=1, k_3=0.7, v_4=0.35, K_4=1, k_5=0.7, v_6=0.35, K_6=1, k_7=0.35, v_8=1, K_8=1, v_c=0.8, K_c=1. The coupling term $Q = KF$ and the mean field F are as defined eq. 11 and 16 in Materials and Methods. A period distribution is generated as described in Materials and Methods, and scaled to obtain a circadian period. We used a nearest neighbor (type 2) coupling. Simulations show that the oscillators synchronize well and are damped in absence of synchronization signals (fig. S3).

One-variable delay model

The one-variable delay model is a limit case of a Goodwin model. The delayed variable $X_\tau \equiv X(t-\tau)$ is the end-product of an infinite linear chain ($\dot{x}_i = x_{i-1} - kx_i$, $i \to \infty$ and $k \to \infty$) representing many post-transcriptional steps that have very short durations. The resulting one-dimensional system displays self-sustained oscillations for a Hill coefficient h high enough. For lower values of h, the system has only a stable steady state.

$$\frac{\mathrm{d}X}{\mathrm{d}t} = \frac{1}{1 + X_\tau^h} - \frac{X}{1+X} + Q + L.$$

We used a value of h = 1.5 and an all-to-all coupling (type 1, $c_0 = 1$). The coupling term is $Q = KF$ and the global (all-to-all) mean field is $F = \langle X \rangle$ (X is not delayed). A period distribution is generated as described in Materials and Methods. Simulations show that the oscillators synchronize well and are damped in absence of periodic synchronization signals (fig. S4).

Leloup-Goldbeter 16-variable model

The Leloup-Goldebeter model of mammalian circadian clock is based on positive and negative regulatory loops involving *Per*, *Cry*, *Bmal1*, *Clock*, and *Rev-Erb*α clock genes. It consists of 16 differential equations describing different forms and cellular localizations of mRNA and protein concentrations (2). The model can give rise to sustained oscillations and reproduce the experimentally observed

phase relationships between mRNA concentrations.

$$\begin{aligned}
\frac{dM_P}{dt} &= v_{stot}v_{sp}\frac{B_n^n}{k_{ap}^n+B_n^n} - v_{mp}\frac{M_P}{k_{mp}+M_P} - k_{dn}M_P + Q + L \\
\frac{dM_C}{dt} &= v_{stot}v_{sc}\frac{B_n^n}{k_{ac}^n+B_n^n} - v_{mc}\frac{M_C}{k_{mc}+M_C} - k_{dn}M_C \\
\frac{dM_B}{dt} &= v_{stot}v_{sb}\frac{k_{ib}^m}{k_{ib}^m+B_n^m} - v_{mb}\frac{M_B}{k_{mb}+M_B} - k_{dn}M_B
\end{aligned}$$

$$\begin{aligned}
\frac{dP_c}{dt} &= k_{sp}M_P - v_{phos}v_{1p}\frac{P_c}{k_p+P_c} + v_{2p}\frac{P_{cp}}{k_{dp}+P_{cp}} + k_4P_{cc} - k_3P_cC_c - k_{dn}P_c \\
\frac{dC_c}{dt} &= k_{sc}M_C - v_{1c}\frac{C_c}{k_p+C_c} + v_{2c}\frac{C_{cp}}{k_{dp}+C_{cp}} + k_4P_{cc} - k_3P_cC_c - k_{dnc}C_c \\
\frac{dP_{cp}}{dt} &= v_{phos}v_{1p}\frac{P_c}{k_p+P_c} - v_{2p}\frac{P_{cp}}{k_{dp}+P_{cp}} - v_{dpc}\frac{P_{cp}}{k_d+P_{cp}} - k_{dn}P_{cp} \\
\frac{dC_{cp}}{dt} &= v_{1c}\frac{C_c}{k_p+C_c} - v_{2c}\frac{C_{cp}}{k_{dp}+C_{cp}} - v_{dcc}\frac{C_{cp}}{k_d+C_{cp}} - k_{dn}C_{cp}
\end{aligned}$$

$$\begin{aligned}
\frac{dP_{cc}}{dt} &= -v_{phos}v_{1pc}\frac{P_{cc}}{k_p+P_{cc}} + v_{2pc}\frac{P_{ccp}}{k_{dp}+P_{ccp}} - k_4P_{cc} + k_3P_cC_c + k_2P_{cn} - k_1P_{cc} - k_{dn}P_{cc} \\
\frac{dP_{cn}}{dt} &= -v_{phos}v_{3pc}\frac{P_{cn}}{k_p+P_{cn}} + v_{4pc}\frac{P_{cnp}}{k_{dp}+P_{cnp}} - k_2P_{cn} + k_1P_{cc} - k_7B_nP_{cn} + k_8I_n - k_{dn}P_{cn} \\
\frac{dP_{ccp}}{dt} &= v_{phos}v_{1pc}\frac{P_{cc}}{k_p+P_{cc}} - v_{2pc}\frac{P_{ccp}}{k_{dp}+P_{ccp}} - v_{dpcc}\frac{P_{ccp}}{k_d+P_{ccp}} - k_{dn}P_{ccp} \\
\frac{dP_{cnp}}{dt} &= v_{phos}v_{3pc}\frac{P_{cn}}{k_p+P_{cn}} - v_{4pc}\frac{P_{cnp}}{k_{dp}+P_{cnp}} - v_{dpcn}\frac{P_{cnp}}{k_d+P_{cnp}} - k_{dn}P_{cnp}
\end{aligned}$$

$$\begin{aligned}
\frac{dB_c}{dt} &= k_{sb}M_B - v_{1b}\frac{B_c}{k_p+B_c} + v_{2b}\frac{B_{cp}}{k_{dp}+B_{cp}} - k_5B_c + k_6B_n - k_{dn}B_c \\
\frac{dB_{cp}}{dt} &= v_{1b}\frac{B_c}{k_p+B_c} - v_{2b}\frac{B_{cp}}{k_{dp}+B_{cp}} - v_{dbc}\frac{B_{cp}}{k_d+B_{cp}} - k_{dn}B_{cp} \\
\frac{dB_n}{dt} &= -v_{3b}\frac{B_n}{k_p+B_n} + v_{4b}\frac{B_{np}}{k_{dp}+B_{np}} + k_5B_c - k_6B_n - k_7B_nP_{cn} + k_8I_n - k_{dn}B_n \\
\frac{dB_{np}}{dt} &= v_{3b}\frac{B_n}{k_p+B_n} - v_{4b}\frac{B_{np}}{k_{dp}+B_{np}} - v_{dbn}\frac{B_{np}}{k_d+B_{np}} - k_{dn}B_{np}
\end{aligned}$$

$$\frac{dI_n}{dt} = -k_8I_n + k_7B_nP_{cn} - v_{din}\frac{I_n}{k_d+I_n} - k_{dn}I_n$$

To obtain damped oscillations, we decreased the Hill coefficients in the first three equations (n from 4 to 1.9 and m from 2 to 1.3). The parameter values are v_{sp} = 1.5, k_{ap} = 0.7, n = 1.9, v_{mp} = 1.1, k_{mp} = 0.31, k_{dn} = 0.01, v_{sc} = 1.1, k_{ac} = 0.6, v_{mc} = 1.0, k_{mc} = 0.4, v_{sb} = 1.0, k_{ib} = 2.2, m = 1.3, v_{mb} = 0.8, k_{mb} = 0.8, k_{sp} = 0.6, v_{1p} = 1.0, k_p = 0.1, v_{2p} = 0.3, k_{dp} = 0.1, k_4 = 0.2, k_3 = 0.4, k_{sc} = 1.6, v_{1c} = 0.6, v_{2c} = 0.1, v_{dpc} = 0.7, v_{dcc} = 0.7, v_{1pc} = 1.0, v_{2pc} = 0.1, k_2 = 0.2, k_1 = 0.4, v_{3pc} = 1.0, v_{4pc} = 0.1, k_7 = 0.5, k_8 = 0.1, v_{dpcc} = 0.7, v_{dpcn} = 0.7, k_{sb} = 0.12, v_{1b} = 0.5, v_{2b} = 0.1, k_5 = 0.4, k_6 = 0.2, v_{dbc} = 0.5, v_{3b} = 0.5, v_{4b} = 0.2, v_{dbn} = 0.6, v_{din} = 0.8, k_{dnc} = 0.12, v_{phos} = 0.4, v_{stot} = 1.0. We used an all-to-all coupling

(type 1, $c_0 = 1$). The coupling term is $Q = KF$ and the global (all-to-all) mean field is $F = \langle M_P \rangle$. A period distribution is generated as described in Materials and Methods. Simulations show that the oscillators synchronize well and are damped in absence of periodic synchronization signals (fig. S5).

References

[1] Gonze D, Bernard S, Waltermann C, Kramer A, Herzel H (2005) Spontaneous synchronization of coupled circadian oscillators. Biophys J 89:120–129.

[2] Leloup JC, Goldbeter A (2003) Toward a detailed computational model for the mammalian circadian clock. Proc Natl Acad Sci USA 100:7051–7056.
