# Supplementary material for: Synchronization-Induced Rhythmicity of Circadian Oscillators in the Suprachiasmatic Nucleus
Source: PLoS Comput Biol. 2007 Apr 13;3(4):e68. doi: 10.1371/journal.pcbi.0030068 (PMC1851983; doi:10.1371/journal.pcbi.0030068)
Supplement: Supplementary file 2 [file pcbi.0030068.sg001.pdf]

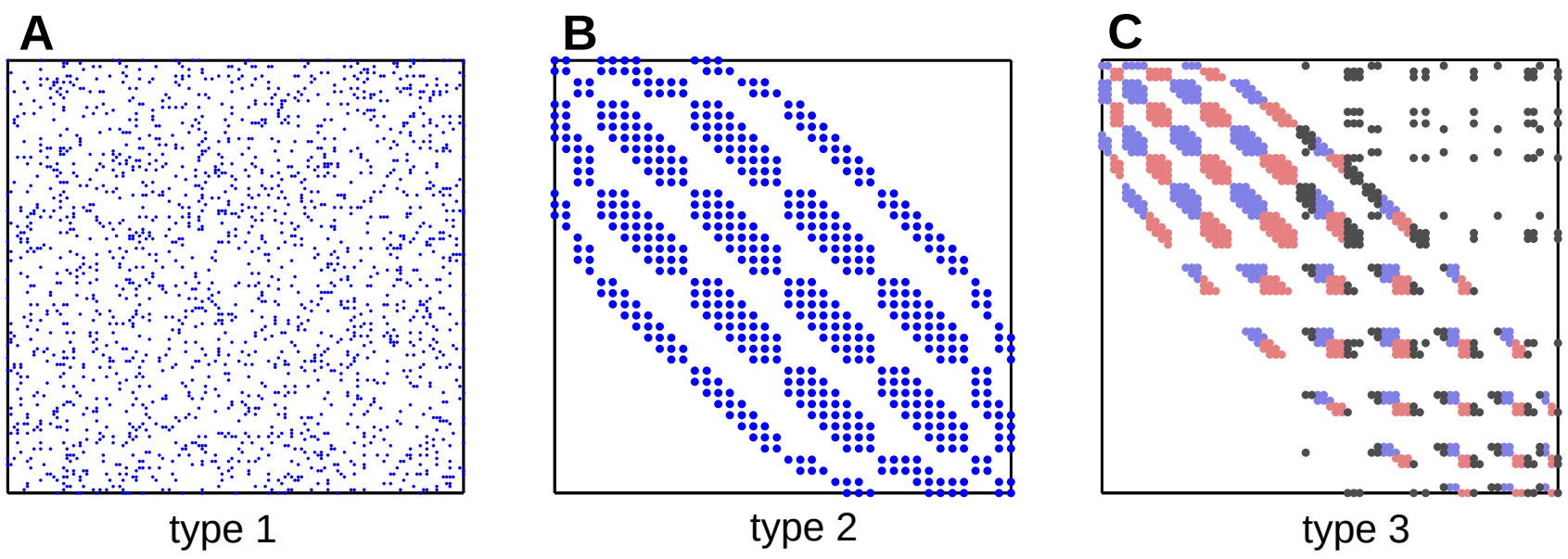

A
B
C
type 1
type 2
type 3
