# Supplementary material for: Synchronization-Induced Rhythmicity of Circadian Oscillators in the Suprachiasmatic Nucleus
Source: PLoS Comput Biol. 2007 Apr 13;3(4):e68. doi: 10.1371/journal.pcbi.0030068 (PMC1851983; doi:10.1371/journal.pcbi.0030068)
Supplement: Supplementary file 3 [file pcbi.0030068.sg002.pdf]

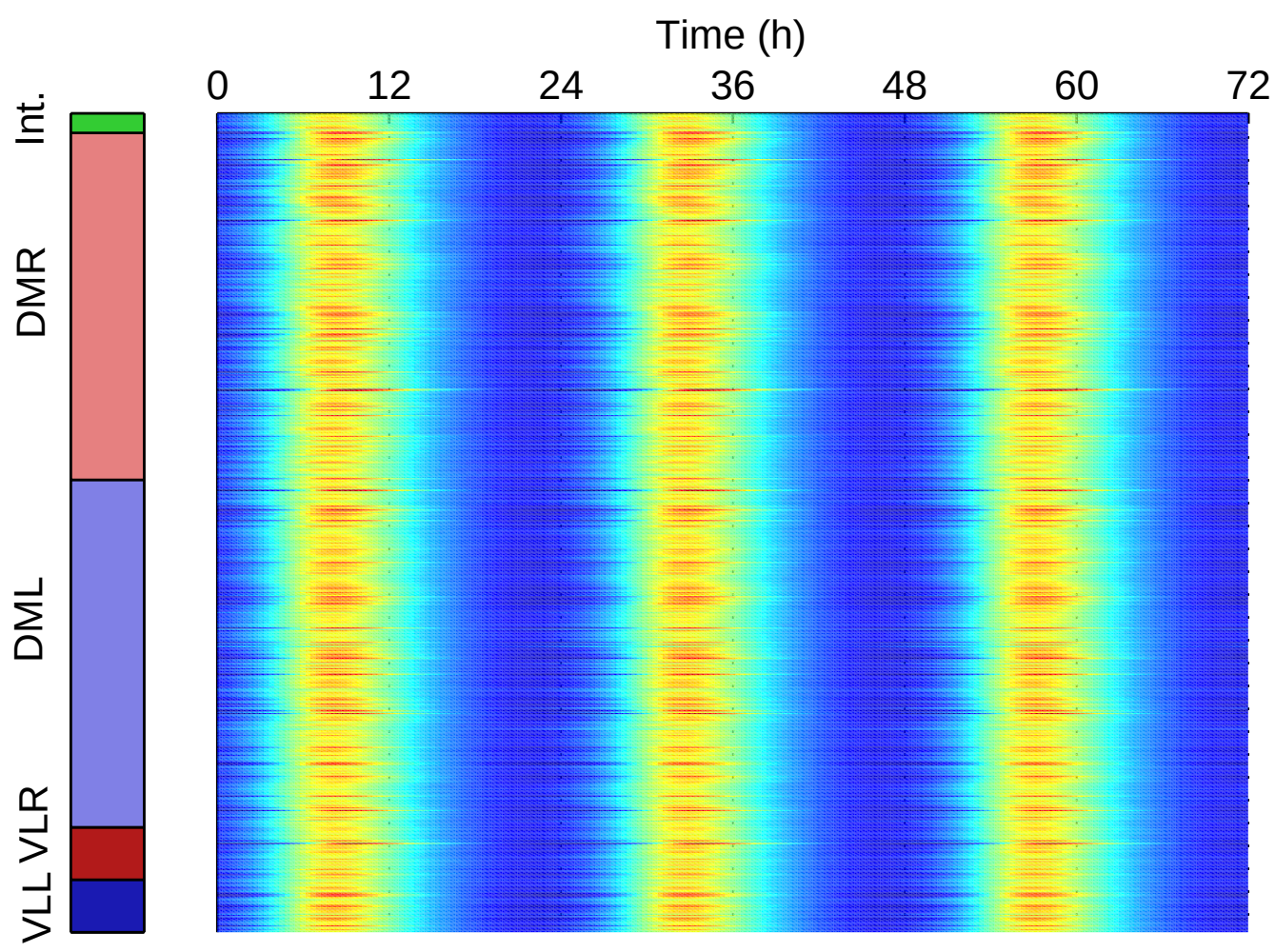
Time (h)
0
12
24
36
48
60
72
Int.
DMR
DML
VLR
VLL
