# Supplementary material for: Synchronization-Induced Rhythmicity of Circadian Oscillators in the Suprachiasmatic Nucleus
Source: PLoS Comput Biol. 2007 Apr 13;3(4):e68. doi: 10.1371/journal.pcbi.0030068 (PMC1851983; doi:10.1371/journal.pcbi.0030068)
Supplement: Supplementary file 4 [file pcbi.0030068.sg003.pdf]

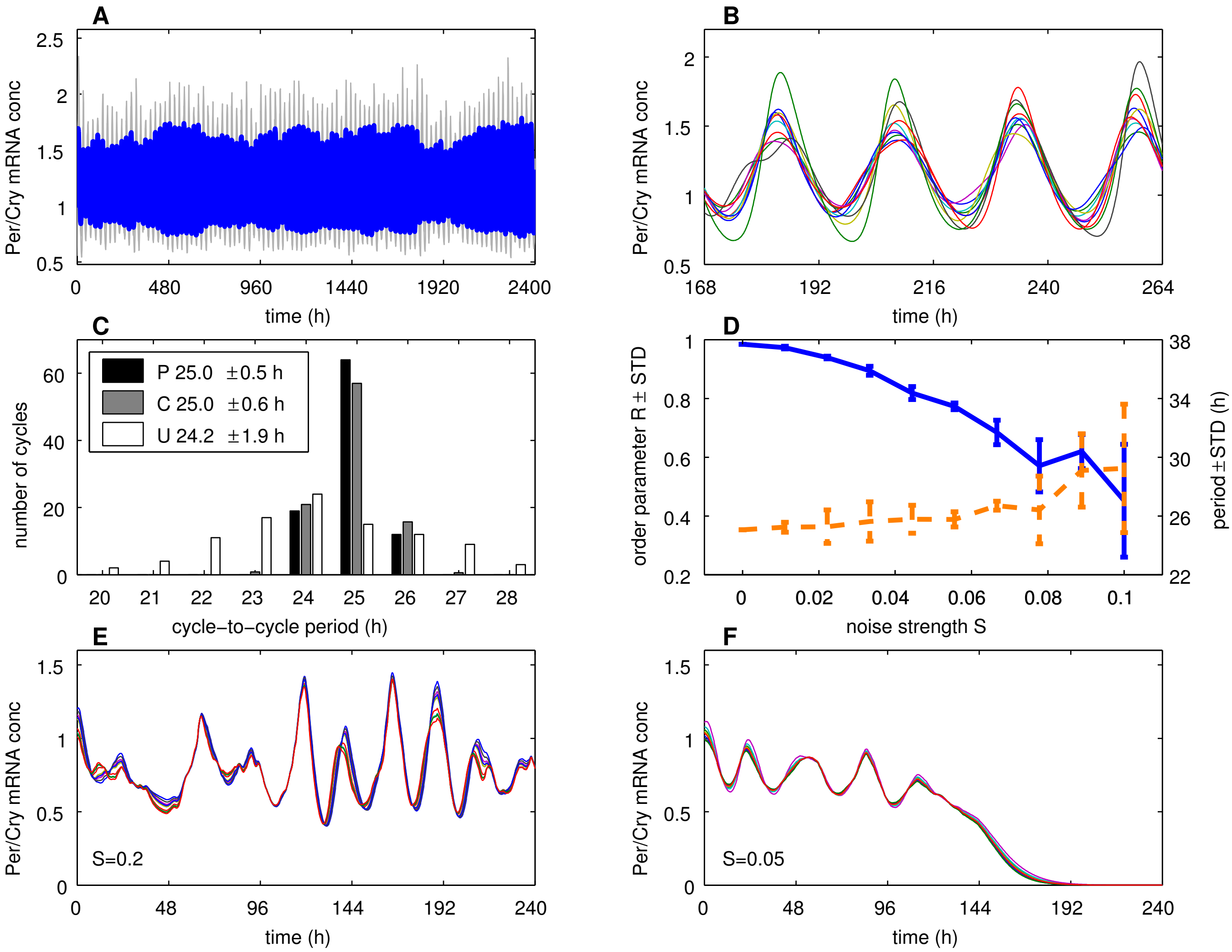

A
Per/Cry mRNA conc
2.5
2
1.5
1
0.5
0
480
960
1440
1920
2400
time (h)
B
Per/Cry mRNA conc
2
1.5
1
0.5
168
192
216
240
264
time (h)
C
number of cycles
P 25.0 ±0.5 h
C 25.0 ±0.6 h
U 24.2 ±1.9 h
60
40
20
0
20
21
22
23
24
25
26
27
28
cycle−to−cycle period (h)
D
order parameter R ± STD
period±STD (h)
1
0.8
0.6
0.4
0.2
38
34
30
26
22
0
0.02
0.04
0.06
0.08
0.1
noise strength S
E
Per/Cry mRNA conc
1.5
1
0.5
0
S=0.2
0
48
96
144
192
240
time (h)
F
Per/Cry mRNA conc
1.5
1
0.5
0
S=0.05
0
48
96
144
192
240
time (h)
