# Supplementary material for: Synchronization-Induced Rhythmicity of Circadian Oscillators in the Suprachiasmatic Nucleus
Source: PLoS Comput Biol. 2007 Apr 13;3(4):e68. doi: 10.1371/journal.pcbi.0030068 (PMC1851983; doi:10.1371/journal.pcbi.0030068)
Supplement: Supplementary file 5 [file pcbi.0030068.sg004.pdf]

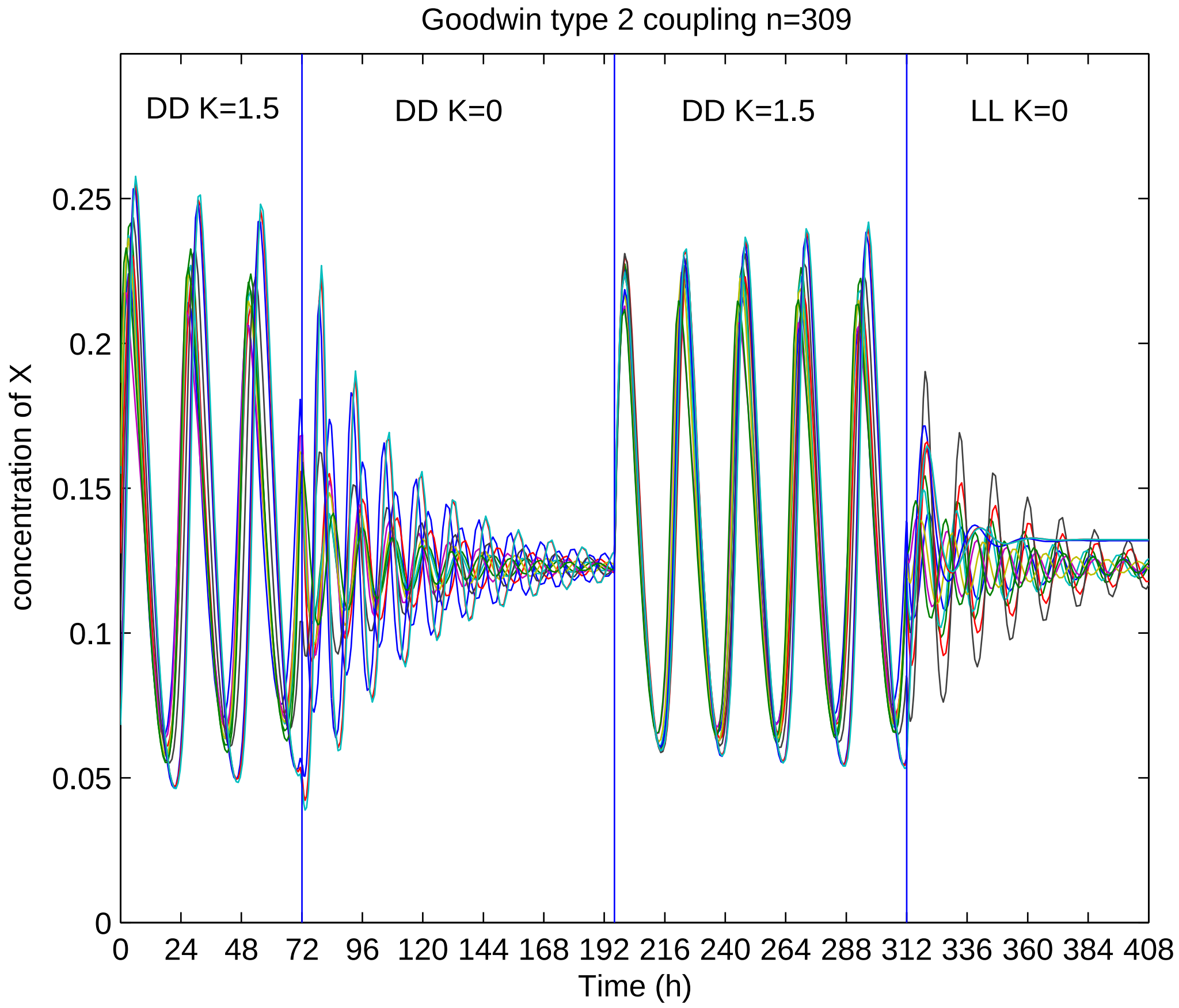
Goodwin type 2 coupling n=309
DD K=1.5
DD K=0
DD K=1.5
LL K=0
concentration of X
0
0.05
0.1
0.15
0.2
0.25
0
24
48
72
96
120
144
168
192
216
240
264
288
312
336
360
384
408
Time (h)
