# Supplementary material for: Synchronization-Induced Rhythmicity of Circadian Oscillators in the Suprachiasmatic Nucleus
Source: PLoS Comput Biol. 2007 Apr 13;3(4):e68. doi: 10.1371/journal.pcbi.0030068 (PMC1851983; doi:10.1371/journal.pcbi.0030068)
Supplement: Supplementary file 6 [file pcbi.0030068.sg005.pdf]

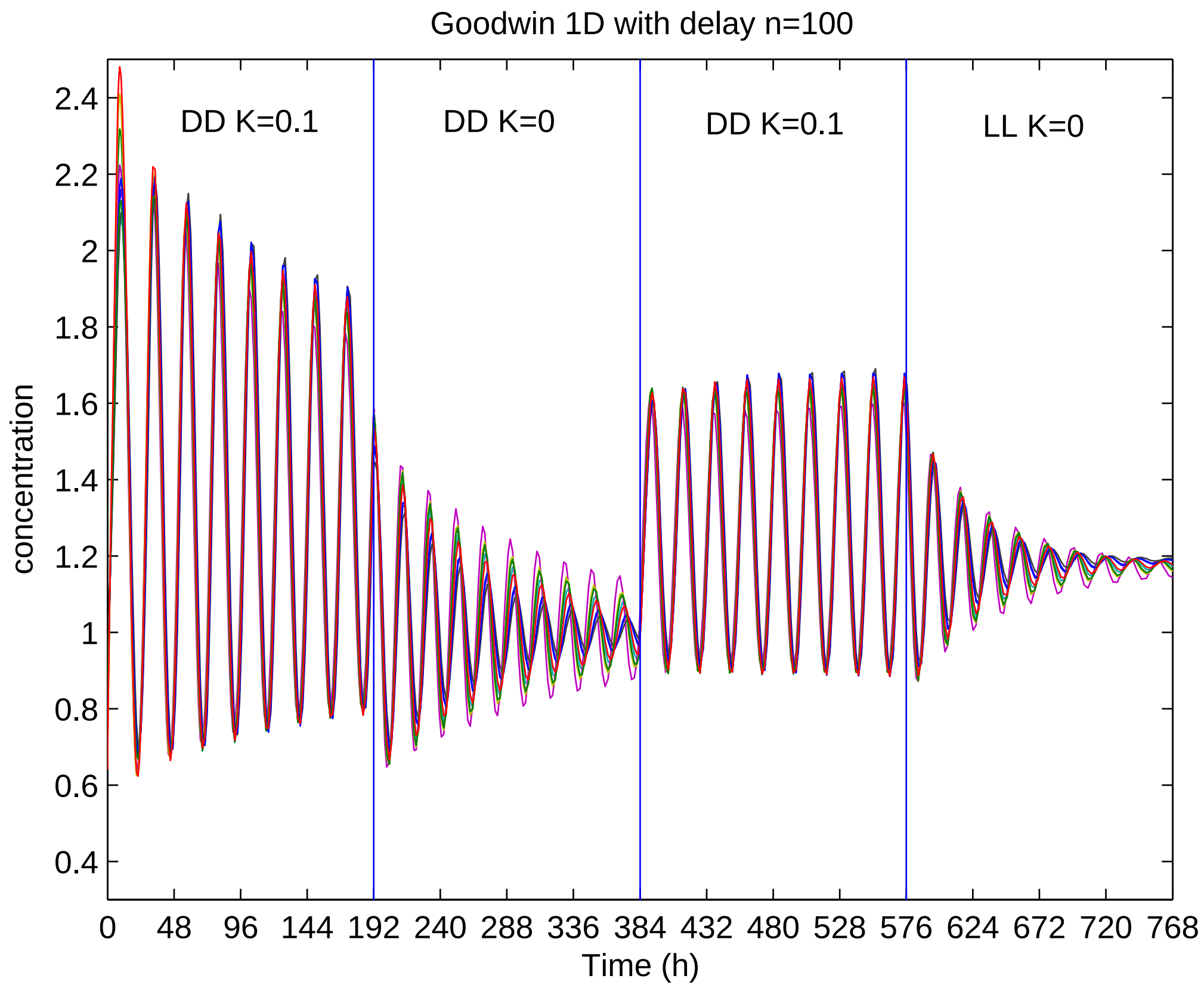
Goodwin 1D with delay n=100
DD K=0.1
DD K=0
DD K=0.1
LL K=0
concentration
2.4
2.2
2
1.8
1.6
1.4
1.2
1
0.8
0.6
0.4
0 48 96 144 192 240 288 336 384 432 480 528 576 624 672 720 768
Time (h)
