# Supplementary material for: Synchronization-Induced Rhythmicity of Circadian Oscillators in the Suprachiasmatic Nucleus
Source: PLoS Comput Biol. 2007 Apr 13;3(4):e68. doi: 10.1371/journal.pcbi.0030068 (PMC1851983; doi:10.1371/journal.pcbi.0030068)
Supplement: Supplementary file 7 [file pcbi.0030068.sg006.pdf]

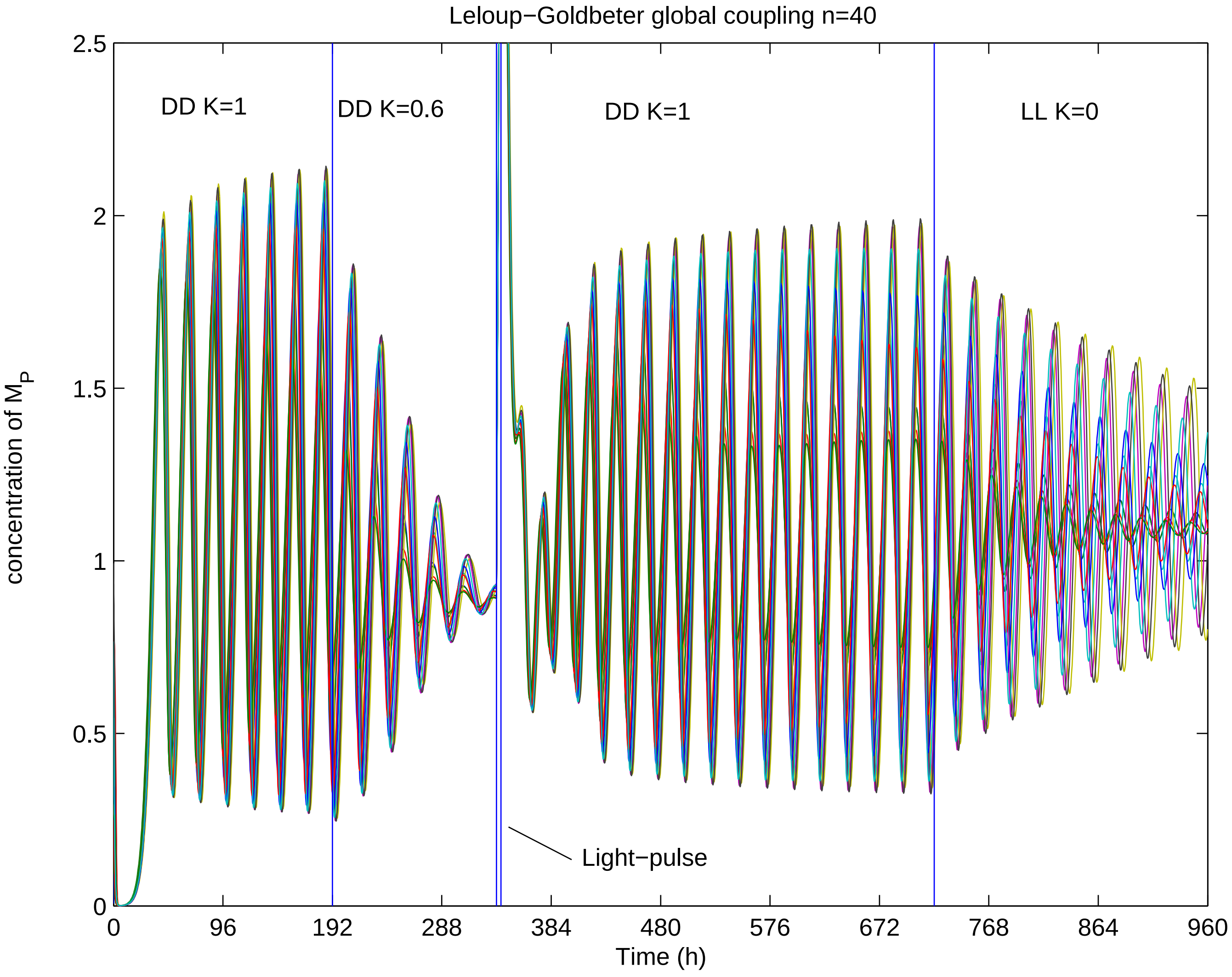
Leloup−Goldbeter global coupling n=40
DD K=1
DD K=0.6
DD K=1
LL K=0
Light−pulse
concentration of M_P
Time (h)
0
96
192
288
384
480
576
672
768
864
960
0
0.5
1
1.5
2
2.5
